# Supplementary material for: Multiple electrolyte derangements among perioperative women with obstructed labour in eastern Uganda: A cross-sectional study
Source: PLOS Glob Public Health. 2023 Jun 12;3(6):e0002012. doi: 10.1371/journal.pgph.0002012 (PMC10259772; doi:10.1371/journal.pgph.0002012)
Supplement: S1 Table — (DOCX) [file pgph.0002012.s001.docx]

**S1_Table: Factors associated with hyperkalaemia and hypokalaemia among women with obstructed labor in eastern Uganda.**

| Variable | Hyperkalaemia  N (%) | COR (95% CI) | AOR (95% CI) | Hypokalaemia  N (%) | | COR (95% CI) | AOR (95% CI) |
| --- | --- | --- | --- | --- | --- | --- | --- |
| Maternal age |  |  |  |  |  | |  |
| ≤19 | 8 (27.6) | 1 |  | 10 (21.3) | 1 | |  |
| 20 to 35 | 20 (69.0) | 1.1 (0.5-2.6) | 1.3 (0.4-3.9) | 31 (65.9) | 1.4 (0.7-3.0) | | 2.2 (0.8-5.4) |
| >35 | 1 (3.4) | 0.5 (0.1-4.3) | 1.2 (0.1-13.3) | 6 (12.8) | 3 (1.0-9.2) | | 5.8 (1.3-25.5) |
| Parity |  |  |  |  |  | |  |
| Primigravida | 18 (62.1) | 1 |  | 25 (53.2) | 1 | |  |
| 2 to 4 | 8 (27.6) | 0.7 (0.3-1.7) | 0.5 (0.2-1.5) | 14 (29.8) | 0.9 (0.5-1.9) | | 0.8 (0.4-1.7) |
| 5+ | 3 (10.3) | 0.6 (0.2-2.2) | 0.5 (0.1-2.7) | 8 (17.0) | 1.3 (0.5-3.0) | | 0.6 (0.2-1.9) |
| Marital status |  |  |  |  |  | |  |
| Single | 6 (20,7) | 1 |  | 8 (17.0) | 1 | |  |
| Married | 23 (79.3) | 0.9 (0.3-2.2) | 0.4 (0.1-1.4) | 39 (83.0) | 1.1 (0.5-2.5) | | 0.8 (0.2-2.5) |
| Religion |  |  |  |  |  | |  |
| Christian | 15 (51.7) | 1 |  | 32 (68.1) | 1 | |  |
| Muslim | 14 (48.3) | 2.1 (1.0-4.6) | 2.8 (1.2-6.5) | 15 (31.9) | 1.0 (0.5-1.9) | | 1.1 (0.5-2.2) |
| Others | 0 (0.0) |  |  | 0 (0.0) |  | |  |
| Education level |  |  |  |  |  | |  |
| None/Primary | 1 4 (48.3) | 1 |  | 25 (53.2) | 1 | |  |
| Secondary/Tertiary | 15 (51.7) | 1.1 (0.5-2.3) | 1.2 (0.4-3.3) | 22 (46.8) | 0.9 (0.5-1.6) | | 0.8 (0.3-1.7) |
| Occupation |  |  |  |  |  | |  |
| Salaried employee | 3 (10.3) | 1 |  | 5 (10.6) | 1 | |  |
| Business | 4 (13.8) | 1.6 (0.3-7.6) | 1.6 (0.3-8.9) | 2 (4.3) | 0.4 (0.1-2.3) | | 0.4 (0.1-2.5) |
| Subsistence Farmer | 5 (17.2) | 1.1 (0.3-5.0) | 1.0 (0.2-5.2) | 7 (14.9) | 0.9 (0.3-3.2) | | 0.9 (0.2-3.4) |
| House wife | 14 (48.3) | 1.0 (0.3-3.6) | 0.8 (0.2-3.4) | 27 (57.4) | 1.2 (0.4-3.2) | | 1.0 (0.3-3.2) |
| Other | 3 (10.3) | 0.7 (0.1-3.7) | 0.3 (0.03-2.0) | 6 (12.8) | 0.9 (0.2-3.0) | | 0.7 (0.1-3.8) |
| Place of residence |  |  |  |  |  | |  |
| Urban | 3 (10.3) | 1 |  | 2 (4.3) | 1 | |  |
| Rural | 26 (89.7) | 1.0 (0.3-3.5) | 0.7 (0.2-2.7) | 45 (95.7) | 2.9 (0.6-12.4) | | 2.7 (0.6-11.9) |
| Alcohol drinking |  |  |  |  |  | |  |
| Yes | 1 (3.4) | 1 |  | 1 (2.1) | 1 | |  |
| No | 28 (96.6) | 0.9 (0.1-7.1) | 0.6 (01-5.6) | 46 (97.9) | 1.5 (0.2-12.1) | | 1.9 (0.2-17.1) |
| HIV status |  |  |  |  |  | |  |
| Positive | 1 (3.4) | 0.6 (0.04-8.7) | 1.0 (0.04-20.6) | 0 (0.0) |  | |  |
| Negative | 26 (89.7) | 0.2 (0.04-1.2) | 0.2 (0.03-1.4) | 47 (100.0) |  | |  |
| Don’t know | 2 (6.9) | 1 |  | 0 (0.0) |  | |  |
| Referred |  |  |  |  |  | |  |
| No | 9 (31.0) | 1 |  | 14 (29.8) | 1 | |  |
| Yes | 20 (69.0) | 1.3 (0.6-2.9) | 1.6 (0.6-4.0) | 33 (70.2) | 1.4 (0.7-2.7) | | 1.4 (0.7-2.9) |
| Herbal medicines use |  |  |  |  |  | |  |
| Yes | 17 (58.6) | 1.1 (0.5-2.4) | 1.2 (0.5-2.8) | 28 (59.6) | 1.2 (0.6-2.1) | | 1.0 (0.5-2.0) |
| No | 12 (41.4) | 1 |  | 19 (43.4) | 1 | |  |
| Labour duration |  |  |  |  |  | |  |
| <12 | 1 (3.4) | 1 |  | 4 (8.5) | 1 | |  |
| 12 to 18 | 6 (20.7) | 3.5 (0.4-30.1) | 5.1 (0.5-50.8) | 12 (25.5) | 1.8 (0.5-6.0) | | 1.7 (0.5-6.3) |
| >18 | 22 (75.9) | 2.7 (0.4-20.6) | 3.8 (0.4-29.3) | 31 (66.0) | 0.9 (0.3-2.7) | | 0.9 (0.3-2.9) |
