# Supplementary material for: Multiple electrolyte derangements among perioperative women with obstructed labour in eastern Uganda: A cross-sectional study
Source: PLOS Glob Public Health. 2023 Jun 12;3(6):e0002012. doi: 10.1371/journal.pgph.0002012 (PMC10259772; doi:10.1371/journal.pgph.0002012)
Supplement: S2 Table — (DOCX) [file pgph.0002012.s002.docx]

**S2_Table: Factors associated with hyponatremia among women with obstructed labor in eastern Uganda**

| Variable | Hyponatremia N (%) | COR (95% CI) | AOR (95% CI) |
| --- | --- | --- | --- |
| Maternal age |  |  |  |
| ≤19 | 20 (28.6) | 1 |  |
| 20 to 35 | 44 (62.9) | 0.9 (0.5-1.7) | 0.9 (0.4-1.9) |
| >35 | 6 (8.6) | 1.4 (0.5-3.8) | 1.3 (0.3-5.1) |
| Parity |  |  |  |
| Primigravida | 39 (55.7) | 1 |  |
| 2 to 4 | 20 (28.6) | 0.8 (0.5-1.5) | 0.8 (0.4-1.6) |
| 5+ | 11 (15.7) | 1.1 (0.5-2.4) | 1.3 (0.3-2.9) |
| Marital status |  |  |  |
| Single | 12 (17.1) | 1 |  |
| Married | 58 (82.9) | 1.1 (0.5-2.2) | 0.8 (0.3-2.3) |
| Religion |  |  |  |
| Christian | 46 (65.7) | 1 |  |
| Muslim | 22 (31.4) | 1.0 (0.6-1.8) | 1.2 (0.7-2.2) |
| Others | 2 (2.9) | 4.7 (0.6-34.4) | 5.4 (0.7-43.9) |
| Education level |  |  |  |
| None/Primary | 35 (50.0) | 1 |  |
| Secondary/Tertiary | 35 (50.0) | 1.1 (0.5-2.3) | 1.0 (0.5-2.0) |
| Occupation |  |  |  |
| Salaried employee | 11 (15.7) | 1 |  |
| Business | 3 (4.3) | 0.2 (0.1-1.0) | 0.3 (0.1-1.0) |
| Subsistence Farmer | 8 (11.4) | 0.4 (0.1-1.1) | 0.3 (0.1-1.0) |
| House wife | 39 (55.7) | 0.7 (0.3-1.5) | 0.6 (0.2-1.4) |
| Other | 9 (12.9) | 0.5 (0.2-1.4) | 0.3 (0.1-1.4) |
| Place of residence |  |  |  |
| Urban | 7 (10.0) | 1 |  |
| Rural | 63 (90.0) | 1.1 (0.5-2.5) | 0.9 (0.4-2.2) |
| Alcohol drinking |  |  |  |
| Yes | 3 (4.3) | 1 |  |
| No | 67 (95.7) | 0.6 (0.2-2.5) | 0.6 (0.1-2.4) |
| HIV status |  |  |  |
| Positive | 1 (3.4) | 1.5 (0.1-15.5) | 1.5 (0.1-19.0) |
| Negative | 26 (89.7) | 0.6 (0.1-3.2) | 0.6 (0.1-3.3) |
| Don’t know | 2 (6.9) | 1 |  |
| Referred |  |  |  |
| No | 21 (30.0) | 1 |  |
| Yes | 49 (70.0) | 1.4 (0.8-2.4) | 1.5 (0.8-2.9) |
| Herbal medicines use |  |  |  |
| Yes | 42 (60.0) | 1.2 (0.7-2.0) | 1.1 (0.6-1.9) |
| No | 28 (40.0) | 1 |  |
| Labour duration |  |  |  |
| <12 | 6 (8.6) | 1 |  |
| 12 to 18 | 12 (17.1) | 1.1 (0.4-3.2) | 1.3 (0.4-4.1) |
| >18 | 52 (74.3) | 1.0 (0.4-2.6) | 1.3 (0.5-3.5) |
