# Supplementary material for: Multiple electrolyte derangements among perioperative women with obstructed labour in eastern Uganda: A cross-sectional study
Source: PLOS Glob Public Health. 2023 Jun 12;3(6):e0002012. doi: 10.1371/journal.pgph.0002012 (PMC10259772; doi:10.1371/journal.pgph.0002012)
Supplement: S3 Table — (DOCX) [file pgph.0002012.s003.docx]

**S3_Table: Factors associated with hypobicarbonatemia among women with obstructed labor in eastern Uganda**

| Variable | Hypobicarbonatemia N (%) | COR (95% CI) | AOR (95% CI) |
| --- | --- | --- | --- |
| Maternal age |  |  |  |
| ≤19 | 93 (27.8) | 1 |  |
| 20 to 35 | 218 (65.3) | 1.1 (0.6-2.1) | 1.1 (0.5-2.5) |
| >35 | 23 (6.9) | 1.4 (0.4-5.2) | 0.7 (0.1-3.8) |
| Parity |  |  |  |
| Primigravida | 181 (54.2) | 1 |  |
| 2 to 4 | 103 (30.8) | 0.8 (0.4-1.4) | 0.8 (0.4-1.7) |
| 5+ | 50 (15.0) | 2.0 (0.7-6.0) | 2.6 (0.7-10.3) |
| Marital status |  |  |  |
| Single | 63 (18.9) | 1 |  |
| Married | 271 (81.1) | 0.8 (0.4-1.8) | 1.3 (0.4-3.4) |
| Religion |  |  |  |
| Christian | 229 (68.6) | 1 |  |
| Muslim | 102 (30.5) | 0.8 (0.4-1.4) | 0.6 (0.3-1.2) |
| Others | 3 (0.9) | 0.4 (0.04-4.4) | 0.3 (0.2-3.1) |
| Occupation |  |  |  |
| Salaried employee | 34 (10.2) | 1 |  |
| Business | 31 (9.3) | 1.3 (0.4-4.4) | 1.5 (0.4-5.8) |
| Subsistence Farmer | 48 (14.4) | 0.8 (0.3-2.1) | 0.7 (0.2-2.3) |
| House wife | 170 (50.9) | 1.4 (0.6-3.5) | 1.9 (0.7-5.1) |
| Other | 51 (15.3) | 2.1 (0.6-7.2) | 3.6 (0.8-16.8) |
| Alcohol drinking |  |  |  |
| Yes | 8 (2.4) | 1 |  |
| No | 326 (97.6) | 3.2 (0.9-11.0) | 3.6 (0.9-14.7) |
| HIV status |  |  |  |
| Positive | 4 (3.4) | 3.3 (0.4-30.7) | 2.8 (0.2-36.6) |
| Negative | 327 (97.9) | 11.4 (2.6-49.0) | **13.0 (2.7-62.6)** |
| Don’t know | 3 (0.9) | 1 |  |
| Referred |  |  |  |
| No | 121 (36.2) | 1 |  |
| Yes | 213 (63.8) | 0.9 (0.5-1.7) | 0.9 (0.4-1.8) |
| Herbal medicines use |  |  |  |
| Yes | 186 (55.7) | 0.8 (0.4-1.4) | 0.8 (0.4-1.6) |
| No | 148 (44.3) | 1 |  |
| Labour duration |  |  |  |
| <12 | 29 (8.6) | 1 |  |
| 12 to 18 | 50 (15.0) | 0.7 (0.2-2.0) | 0.5 (0.1-1.8) |
| >18 | 255 (76.4) | 1.2 (0.4-3.3) | 1.1 (0.4-3.4) |
