# Supplementary material for: Multiple electrolyte derangements among perioperative women with obstructed labour in eastern Uganda: A cross-sectional study
Source: PLOS Glob Public Health. 2023 Jun 12;3(6):e0002012. doi: 10.1371/journal.pgph.0002012 (PMC10259772; doi:10.1371/journal.pgph.0002012)
Supplement: S4 Table — (DOCX) [file pgph.0002012.s004.docx]

**S4_Table: Factors associated with hypocalcaemia among women with obstructed labor in eastern Uganda**

| Variable | Hypocalcaemia N (%) | COR (95% CI) | AOR (95% CI) |
| --- | --- | --- | --- |
| Maternal age |  |  |  |
| ≤19 | 30 (26.6) | 1 |  |
| 20 to 35 | 75 (66.4) | 1.1 (0.7-1.9) | 1.2 (0.7-2.2) |
| >35 | 8 (7.1) | 1.2 (0.5-3.0) | 1.0 (0.3-3.4) |
| Parity |  |  |  |
| Primigravida | 61 (53.9) | 1 |  |
| 2 to 4 | 33 (29.2) | 0.9 (0.5-1.4) | 0.9 (0.5-1.5) |
| 5+ | 19 (16.8) | 1.3 (0.7-2.5) | 1.4 (0.6-3.3) |
| Marital status |  |  |  |
| Single | 23 (20.4) | 1 |  |
| Married | 90 (79.7) | 0.8 (0.5-1.5) | 0.7 (0.3-1.6) |
| Religion |  |  |  |
| Christian | 78 (69.0) | 1 |  |
| Muslim | 35 (31.0) | 1.0 (0.6-1.5) | 0.9 (0.6-1.5) |
| Others | 0 (0.0) | - | - |
| Occupation |  |  |  |
| Salaried employee | 13 (11.5) | 1 |  |
| Business | 6 (5.3) | 0.4 (0.1-1.3) | 0.4 (0.1-1.4) |
| Subsistence Farmer | 23 (20.4) | 1.3 (0.6-3.0) | 1.5 (0.6-3.9) |
| House wife | 54 (47.8) | 0.8 (0.4-1.7) | 0.9 (0.4-2.0) |
| Other | 17 (15.0) | 0.9 (0.4-2.2) | 0.8 (0.3-2.5) |
| Alcohol drinking |  |  |  |
| Yes | 1 (0.9) | 1 |  |
| No | 112 (99.1) | 4.6 (0.6-36.4) | 4.5 (0.5-37.3) |
| HIV status |  |  |  |
| Positive | 1 (0.9) | 0.3 (0.3-4.4) | 0.4 (0.02-5.3) |
| Negative | 109 (96.5) | 0.6 (0.2-2.9) | 0.6 (0.1-2.8) |
| Don’t know | 3 (2.7) | 1 |  |
| Referred |  |  |  |
| No | 41 (36.3) | 1 |  |
| Yes | 72 (63.7) | 1.0 (0.6-1.5) | 0.8 (0.5-1.3) |
| Herbal medicines use |  |  |  |
| Yes | 71 (62.8) | 1.4 (0.9-2.3) | 1.6 (1.0-2.6) |
| No | 42 (37.2) | 1 |  |
| Labour duration |  |  |  |
| <12 | 6 (5.3) | 1 |  |
| 12 to 18 | 20 (17.7) | 2.2 (0.8-6.1) | 2.3 (0.8-6.7) |
| >18 | 87 (77.0) | 2.0 (0.8-5.0) | 1.7 (0.7-4.5) |
