# Supplementary material for: Multiple electrolyte derangements among perioperative women with obstructed labour in eastern Uganda: A cross-sectional study
Source: PLOS Glob Public Health. 2023 Jun 12;3(6):e0002012. doi: 10.1371/journal.pgph.0002012 (PMC10259772; doi:10.1371/journal.pgph.0002012)
Supplement: S5 Table — (DOCX) [file pgph.0002012.s005.docx]

**S5_Table: Factors associated with perinatal death among women with obstructed labour in eastern Uganda**

| **Variable** |  | **COR (95% CI)** | **P-value** | **AOR (95% CI)** | **P-value** |
| --- | --- | --- | --- | --- | --- |
| **Multiple electrolyte derangements** |  |  |  |  |  |
| No |  | 1 |  | 1 |  |
| Yes |  | 1.5 (0.8-2.9) | 0.251 | 2.1 (0.9-4.7) | 0.072 |
| **Maternal age** |  |  |  |  |  |
| ≤19 |  | 1 |  | 1 |  |
| 20-35 |  | 0.6 (0.3-1.2) | 0.162 | 0.5 (0.2-1.4) | 0.183 |
| >35 |  | 1.2 (0.4-4.0) | 0.773 | 0.5 (0.1-3.8) | 0.510 |
| **Parity** |  |  |  |  |  |
| Primigravida |  | 1 |  | 1 |  |
| 2 to 4 |  | 1.1 (0.5-2.3) | 0.791 | 1.9 (0.7-5.3) | 0.200 |
| 5+ |  | 1.7 (0.7-4.0) | 0.259 | 2.4 (0.6-9.6) | 0.205 |
| **Referral status** |  |  |  |  |  |
| No |  | 1 |  | 1 |  |
| Yes |  | 3.7 (1.5-9.1) | 0.004 | **6.4 (1.8-22.6)** | **0.004** |
| **Height of mother** |  |  |  |  |  |
|  |  | 0.95 (0.9-0.98) | 0.005 | **0.94 (0.9-1.0)** | **0.008** |
| **Weight of mother** |  |  |  |  |  |
|  |  | 0.97 (0.9-1.0) | 0.110 | 1.0 (0.95-1.0) | 0.696 |
