# Supplementary material for: Multiple electrolyte derangements among perioperative women with obstructed labour in eastern Uganda: A cross-sectional study
Source: PLOS Glob Public Health. 2023 Jun 12;3(6):e0002012. doi: 10.1371/journal.pgph.0002012 (PMC10259772; doi:10.1371/journal.pgph.0002012)
Supplement: Attachment — Submitted filename: Rebuttal letter 01_MD.docx [file pgph.0002012.s007.docx]

**Re; Submission ID PGPH-D-22-01944 “Multiple electrolyte derangements among perioperative women with obstructed labour in eastern Uganda: A cross-sectional study”**

Thank you for revising our manuscript.

Below is our point-by-point response to each comment.

Thank you!

| **Comment** | **Response to comment** | **Line number** |
| --- | --- | --- |
|  | | |
| Editor | | |
| 1. Please amend your detailed Financial Disclosure statement. This is published with the article. It must therefore be completed in full sentences and contain the exact wording you wish to be published  State the initials, alongside each funding source, of each author to receive each grant. | Thank you, there is no specific funding for this secondary analysis but the primary study was supported by Survival Pluss Project at Makerere University. Funded by NORHED under NORAD. UGA-13-0030, Prof. James K. Tumwine. The funders had no role in study design, data collection and analysis, decision to publish, or preparation of the manuscript. |  |
| 2. Please provide separate figure files in .tif or .eps format. | Thank you, this has been done. |  |
| 3. We have noticed that you have uploaded Supporting Information files, but you have not included a list of legends. Please add a full list of legends for your Supporting Information files after the references list. | Thank you, this has been rectified. | 466 – 475 |
| 4. In the online submission form, you indicated that "Data supporting the results reported in this manuscript can be obtained from the corresponding author on request.". All PLOS journals now require all data underlying the findings described in their manuscript to be freely available to other researchers, either 1. In a public repository, 2. Within the manuscript itself, or 3. Uploaded as supplementary information. | Thank you for this observation, the dataset that has been used for this analysis has been uploaded as supplementary information. |  |
| Reviewer #1 |  |  |
| Good paper to emphasize the life threatening electrolyte derangement which are common among women with obstructed labour in the perioperative period. However, the study had some limitations by means of the lack of a comparison group of women who laboured successfully without obstruction makes it difficult to attribute these electrolyte derangements to obstructed labour. | Thank you for this comment. |  |
| Reviewer #2 |  |  |
| 1. The study reported by the authors add to the literature what is already known about the effect of protracted labour on electrolyte levels. Since the baseline data as reported by the authors are already known in the literature, one would have expected that the authors would have considered extending the what is known about the subject by including at least birth outcomes to try to link the electrolyte derangements to maternal and fetal outcomes. However, this was not done even though the authors had ample time to have collected such data. If such data is available, authors should include this in the revised manuscript to bring out some novelty to the data presentation. The authors write on page 17 “. This information is of clinical importance because prolonged uncorrected metabolic acidosis is known to cause low fetal pH, low, Apgar scores and asphyxia in the newborn.” This is suggestive that maternal and/or fetal outcomes are dependent on these electrolyte levels. Thus, why the authors did not include such data collection is not so clear. | Thank you for this suggestion, we have gone back to our data and these details have been added. | 256 – 264 |
| 2. Since the most prevalent electrolyte disorder was hypobicarbonatemia, as reported by the authors on page 16, “Hypobicarbonatemia was the most prevalent electrolyte disorder, more than three quarters (¾) of the participants had a low median blood bicarbonate level of 12 mmol/L”, the authors should at least consider regression analyses for factors associated with this electrolyte derangement since it is most pronounced. | Thank you for this comment, we have conducted the regression analyses for factors associated with hypobicarbonatemia as requested and it’s indicated in the supplementary file |  |
| 3. Also, since about one third of our participants had a hypocalcaemia (total calcium), author should consider predisposition to this as well. | Thank you, this has been done and added to the supplementary file. |  |
| 4. Also, could the authors consider including as supplementary data analyses of factors predictive of participants having 3, 4, 5 or 6 electrolyte derangements? This would be an important addition since there might be reason(s) why one person may develop combinations of these electrolyte imbalances. | Thank you for this comment, we have conducted a regression comparing those who had none or one electrolyte imbalance and those who had two or more electrolyte imbalances (Table 3) | 265 – 273 |
| Reviewer #3 |  |  |
| You state that hypocalcemia (total calcium) is common in your population. Is this possibly related to a pre-eclamptic condition of some of the mothers in your cohort? Also, labouring women (and even more so women with obstructed labour) present polypnea. Can this be related to observed calcium derangements? Please comment. | Thank you for this comment. Indeed, pre-eclampsia is associated with hypocalcaemia. However, we excluded all women that had pre-eclampsia. |  |
| Factors listed in line 321-326 represent strong limitations to this study, and must be discussed in the limitation section. | Thank you for pointing this out, we have discussed this in the limitation section. | 364 – 366 |
| A thorough English mothertongue revision should also be performed before resubmission. | Thank you, this has been done. |  |
