# Supplementary material for: Multiple electrolyte derangements among perioperative women with obstructed labour in eastern Uganda: A cross-sectional study
Source: PLOS Glob Public Health. 2023 Jun 12;3(6):e0002012. doi: 10.1371/journal.pgph.0002012 (PMC10259772; doi:10.1371/journal.pgph.0002012)
Supplement: Attachment — Submitted filename: REBUTTAL 140423.docx [file pgph.0002012.s008.docx]

**Re; Submission ID PGPH-D-22-01944 “Multiple electrolyte derangements among perioperative women with obstructed labour in eastern Uganda: A cross-sectional study”**

Thank you for revising our manuscript.

Below is our point-by-point response to each comment.

Thank you!

| **Comment** | **Response to comment** | **Line number** |
| --- | --- | --- |
|  | | |
| Reviewer #3 | | |
| 1. I can't find in the revised manuscript a discussion addressing the points I raised in my previous review. Is hypocalcemia  possibly due to a pre-eclamptic condition? If yes, in what percentage of the set of patients presented by authors? Also, is hypocalcemia related to labouring polypnea of women? Please comment. | Thank you very much for this observation. Yes, hypocalcaemia could be as a result of pre-eclampsia. However, we excluded all mothers that had pre-eclampsia.  Also, hypocalcaemia in labour could be attributed to labouring polypnea. However, in our study almost all (90%) participants had a normal respiratory rate. | 128-131  340-342 |
